# Potential adverse effects of botanical supplementation in high-fat-fed female mice

**DOI:** 10.1186/s13293-018-0199-1

**Published:** 2018-09-12

**Authors:** Scott Fuller, Yongmei Yu, Tamra Mendoza, David M. Ribnicky, William T. Cefalu, Z. Elizabeth Floyd

**Affiliations:** 1 Pennington Biomedical Research Center, Louisiana State University System, Baton Rouge, LA 70808 USA; 2 School of Kinesiology, University of Louisiana at Lafayette, Lafayette, LA 70506 USA; 3 Biotech Center, Rutgers University, New Brunswick, NJ 08901 USA

**Keywords:** Sex, Botanical, Skeletal muscle, Insulin, Metabolic syndrome, Liver, Obesity

## Abstract

**Background:**

Insulin resistance underlies metabolic syndrome and is associated with excess adiposity and visceral fat accumulation, which is more frequently observed in males than females. However, in young females, the prevalence of metabolic syndrome is rising, mainly driven by accumulation of abdominal visceral fat. The degree to which sex-related differences could influence the development of insulin resistance remains unclear, and studies of potential therapeutic strategies to combat metabolic syndrome using rodent models have focused predominantly on males. We therefore evaluated the effects of two nutritional supplements derived from botanical sources, an extract of *Artemisia dracunculus* L. (termed PMI5011) and *Momordica charantia* (commonly known as bitter melon), on female mice challenged with a high-fat diet in order to determine if dietary intake of these supplements could ameliorate obesity-induced insulin resistance and metabolic inflexibility in skeletal muscle.

**Methods:**

Body composition, physical activity and energy expenditure, fatty acid oxidation, insulin signaling, and gene and protein expression of factors controlling lipid metabolism and ectopic lipid accumulation were evaluated in female mice fed a high-fat diet supplemented with either PMI5011 or bitter melon. Statistical significance was assessed by unpaired two-tailed *t* test and repeated measures ANOVA.

**Results:**

PMI5011 supplementation resulted in increased body weight and adiposity, while bitter melon did not induce changes in these parameters. Pyruvate tolerance testing indicated that both supplements increased hepatic glucose production. Both supplements induced a significant suppression in fatty acid oxidation in skeletal muscle homogenates treated with pyruvate, indicating enhanced metabolic flexibility. PMI5011 reduced lipid accumulation in skeletal muscle, while bitter melon induced a downward trend in lipid accumulation in the skeletal muscle and liver. This was accompanied by transcriptional regulation of autophagic genes by bitter melon in the liver.

**Conclusions:**

Data from the current study indicates that dietary supplementation with PMI5011 and bitter melon evokes a divergent, and generally less favorable, set of metabolic responses in female mice compared to effects previously observed in males. Our findings underscore the importance of considering sex-related variations in responses to dietary supplementation aimed at combating metabolic syndrome.

**Electronic supplementary material:**

The online version of this article (10.1186/s13293-018-0199-1) contains supplementary material, which is available to authorized users.

## Background

The prevalence of metabolic syndrome (MetS) has risen to epidemic proportions in recent decades and constitutes an emergent threat to global public health [1–3]. MetS is characterized by abdominal obesity, insulin resistance, dyslipidemia, and hypertension; these factors predispose individuals to greater risk for cardiovascular disease, chronic kidney disease, and several types of cancer [4–7]. Although research effort focused on MetS continues to intensify as the disease burden associated with the condition becomes ever more acute, an issue that remains relatively unclear is the extent to which MetS differentially affects males versus females [8, 9]. In light of evidence that there are sex-related differences in the prevalence and pathophysiology of MetS, research specifically evaluating MetS in females offers the potential for developing sex-specific treatment modalities for chronic diseases including obesity, cardiovascular, and metabolic diseases [10, 11]. However, the research undertaken thus far in both human and animal models has focused primarily on males [8]. Thus, there remains a relatively underserved need for investigation into MetS specifically in females.

Current recommendations for the prevention and treatment of MetS emphasize lifestyle modifications that include dietary changes, weight loss, and exercise [12]. However, despite persistent advice to the public emphasizing the importance of lifestyle factors in the prevention and management of MetS, the rising prevalence of the syndrome and its associated pathologies is a testament to the difficulties encountered in successfully implementing long-term behavioral changes. Pharmacotherapy has been employed with varying degrees of success, although disadvantages associated with prolonged use of pharmaceuticals include side effects, cost, and public access [13, 14], which is a particularly acute problem in the developing world where the incidence of MetS is escalating at an alarming rate and availability of pharmaceuticals can be a limiting factor [15]. Natural products derived from food sources therefore represent an attractive complementary therapy for the treatment of MetS due to their relative safety and tolerability compared to several of the drugs currently available, although vigilance remains necessary to ensure proper safety standards for nutritional supplements in the marketplace [16, 17].

Several species of the plant genus *Artemisia* have demonstrated potential for ameliorating MetS in laboratory studies [18–20]. In particular, *Artemisia dracunculus* L., or Russian tarragon, is a perennial herb with a documented history of medicinal use as an anti-diabetic [21]. Previous studies in our laboratory provide evidence that an ethanolic extract of *A*. *dracunculus* L. termed PMI5011 favorably modulates insulin signaling, lipid metabolism, and glucose homeostasis primarily via effects on skeletal muscle both in vitro and in obese male mice with established insulin resistance [22–25]. We recently extended these findings by demonstrating that early dietary supplementation with PMI5011 in male mice protects against the development of insulin resistance and ectopic lipid accumulation in the skeletal muscle and liver independent of any changes in adiposity or body mass [26]. *Momordica charantia*, commonly known as bitter melon, has been demonstrated to have anti-hyperglycemic and hypolipidemic [27, 28] effects and is a staple of the traditional diet in Okinawa, where rates of mortality and morbidity due to chronic diseases are among the lowest in the world [29]. Evidence from mechanistic studies in diabetic rodents indicates that bitter melon enhances insulin sensitivity by decreasing serum levels of the pro-inflammatory modulators tumor necrosis factor-alpha (TNF-α) and interleukin-6 (IL-6), decreasing expression of suppressor of cytokine signaling-3 (SOCS-3) and c-Jun N-terminal kinase (JNK), and augmenting insulin-stimulated tyrosine phosphorylation of the insulin receptor substrate-1 (IRS-1) [27, 30]. Multiple studies in male rodents from our laboratories and others demonstrate that bioactives in bitter melon improve insulin sensitivity, possibly via reduced skeletal muscle and hepatic lipid accumulation [31–33]. The effect of bitter melon extract on hepatic lipids is attributed to reduced glucose production and lipid synthesis in the liver [34].

While experimental evidence demonstrates that PMI5011 and bitter melon favorably modulate insulin responsiveness and lipid metabolism in male rodents [26, 35], sex differences in the prevalence and pathogenesis of MetS raise the possibility that females might respond differently to dietary intervention. Given the current scarcity of data in females, we sought to evaluate the effectiveness of PMI5011 and bitter melon in female mice in order to clarify whether sex differences in the response to botanical dietary supplementation might be evident. In the present study, we evaluated the hypothesis that dietary supplementation with PMI5011 or bitter melon prior to the onset of high-fat diet-induced obesity prevents development of high-fat diet-related insulin resistance in female C57BL/6 mice. The novel results reported herein indicate that female mice respond in a generally less favorable manner to supplementation with PMI5011 and bitter melon compared to the pattern previously reported in males [26]. The findings of the present study therefore indicate that sex is an important biological variable that merits serious consideration when evaluating the safety and efficacy of dietary interventions aimed at combating metabolic syndrome.

## Methods

### Sourcing and characterization of PMI5011 extract

The PMI5011 botanical extract from *Artemisia dracunculus* L. was provided by the Botanical and Dietary Supplement Research Center at Pennington Biomedical Research Center. Bitter melon was obtained from Verdure Sciences (Noblesville, IN). Detailed information about quality control, preparation, and biochemical characterization of PMI5011 has been previously reported [24, 25, 36–40].

### Experimental animals

Reproductively intact female C57BL/6J mice were obtained from Jackson Laboratories (Bar Harbor, ME). The estrous cycle was not evaluated at the end of the 4-month study as evidence shows estrous cycle stage does not significantly contribute to variability of molecular or metabolic outcomes measured in female mice [41]. All animal experiments were approved by the Pennington Biomedical Research Center Animal Care and Use Committee (protocol #922). The animals were singly housed with a 12-h light-dark cycle at 24 °C. At 4 weeks of age, mice of similar body weight were randomly assigned (*n* = 14/group) to a defined low-fat diet (LFD; 10% kcal fat, Research Diets, #D12450H) or the low-fat diet supplemented with 1% *w*/*w* PMI5011 or bitter melon. After 4 weeks, the LFD only-fed mice were switched to a high-fat diet (HFD; 45% kcal fat, Research Diets, #D12451) and maintained as the control group. The mice fed a LFD supplemented with either PMI5011 or bitter melon were switched to the HFD supplemented with PMI5011 or bitter melon, formulated with the same mass botanical extract/kcal (equivalent to 1.2% *w*/*w* HFD) as contained in the LFD and were fed ad libitum for 3 months thereafter. The 45% fat content is similar to the fat intake (30–40% of energy intake) for adult men and women in the USA [42]. Body weight and food intake were measured weekly, and body composition was measured bi-weekly by nuclear magnetic resonance (Bruker, Billerica, MA). Activity, food intake, and indirect calorimetry were measured at 12 weeks on each diet (TSE PhenoMaster). The mice were acclimated to the TSE chambers for 2 days prior to data collection over 4 days. At the end of the study, the mice were euthanized between 7 and 11 AM. Human insulin (Humulin, Eli Lilly, Indianapolis, IN) was administered to a subgroup of the control and botanical-supplemented mice (7/group) at a dose of 1.5 U/kg 10 min prior to euthanasia to assay insulin signaling.

### Glucose and insulin tolerance tests

For the glucose (GTT) and insulin (ITT) tolerance tests, the amount of glucose or insulin administered was normalized to body weight, which did not vary significantly among groups (23.0∓ 0.57 g body weight for females) at 10 weeks on the HFDs. Female mice were fasted 4 h prior to administering 2 g/kg body weight of glucose/mouse (GTT) or 1 U/kg body weight of insulin/mouse (HumulinR) (ITT) by intraperitoneal injection.

### Blood chemistry

Fasting glucose levels were measured in whole blood using a Breeze2 glucometer (Bayer, Leverkusen, Germany). Fasting insulin levels were assayed via ELISA (Crystal Chem, Downers Grove, IL). Serum nonesterified fatty acids (Abcam, Cambridge, MA), triglycerides (Eagle Diagnostics, Cedar Hill, TX), and total cholesterol (Cell Biolabs, San Diego, CA) levels were assayed according to manufacturers’ instructions. The index of homeostasis model assessment for insulin resistance, e.g., HOMA-IR [insulin (mU/L) × glucose (mM)/22.5], of each animal was calculated from fasting glucose and insulin levels [43]. Triglyceride levels in the skeletal muscle and liver were assayed according to Folch et al. [44] and reported as milligrams per deciliter. Visible fat was carefully removed from the tissue before assaying triglyceride levels.

### Immunohistochemistry

A portion of mixed gastrocnemius muscle and liver was fixed in 10% formalin, embedded in paraffin, and sectioned onto slides. The sections were hematoxylin and eosin (H&E) stained and scanned (NanoZoomer Digital Pathology, Hamamatsu Corp., Bridgewater, NJ).

### Fatty acid oxidation assay

Mixed gastrocnemius muscle homogenates were prepared as described [45]. Palmitate oxidation was assessed in the whole muscle homogenates as described by Hulver et al [46] with ^14^CO_2_ collected over 60 min. When present, pyruvate was added at a final concentration of 10 mM. CO_2_ levels were normalized to total protein and palmitate oxidation reported as nmol CO_2_/mg protein/h.

### Analysis of protein expression

Skeletal muscle and liver lysates were prepared from powdered tissue by homogenizing in 25 mM HEPES, pH 7.4, 1% Igepal CA630, 137 mM NaCl, 1 mM PMSF, 10 μg/ml aprotinin, 1 μg/ml pepstatin, 5 μg/ml leupeptin, 10 mM Na_4_P_2_O_7_, 100 mM NaF, and 2 mM NaVO_4_ using a Sonifier 450 homogenizer (VWR, Radnor, PA). The samples were centrifuged at 14,000×*g* for 10 min at 4 °C. Protein concentrations were determined using a BCA assay (Thermo Fisher Scientific, Rockford, IL) according to the manufacturer’s instructions. The tissue supernatants (50 μg) were resolved by SDS-PAGE and subjected to immunoblotting using chemiluminescence detection (Thermo Fisher Scientific, Rockford, IL) and quantified as described [47]. Nitrocellulose membranes were incubated with antibodies for 1–2 h at room temperature or overnight at 4 °C as indicated. An additional file provides detailed information about each antibody used (see Additional file 1).

### Analysis of gene expression

Total RNA was purified from powdered the skeletal muscle tissue or liver using Direct-zol RNA MiniPrep (ZYMO Research, Irvine, CA). In each case, RNA (500 ng) was reverse transcribed using Multiscribe Reverse Transcriptase (Applied Biosystems, Thermo Fisher Scientific, Waltham, MA) with random primers at 37 °C for 2 h. Real-time PCR was performed with PowerUP SYBR Green Master Mix (Applied Biosystems) according to the manufacturer’s instructions, using the 7900 Real-Time PCR system and universal cycling conditions (50 °C for 2 min; 95 °C for 10 min; 40 cycles of 95 °C for 15 s and 60 °C for 1 min; followed by 95 °C for 15 s, 60 °C for 15 s, and 95 °C for 15 s). The assays were performed in triplicate, and the results were normalized to *Cyclophilin B* mRNA and analyzed using the 2^−ΔΔCT^method with the control diet used as the calibrator. The gene list is provided in an additional file (see Additional file 2).

### Statistical analysis

Normal distribution of the data for glucose and insulin levels, food intake, and body weight was determined using the D’Agostino-Pearson omnibus K2 normality test. Western blot data was quantified using Un-Scan-It software (version 3, Silk Scientific). Statistical significance was determined using an unpaired two-tailed *t* test or repeated measures ANOVA. All statistical analysis was carried out using JMP Pro13 (SAS Institute) and GraphPad Prism 5 software (GraphPad Software, La Jolla, CA). Variability is expressed as the mean ± standard deviation.

## Results

### Effect of PMI5011 and bitter melon on body composition in female mice

**Fig. 1:**
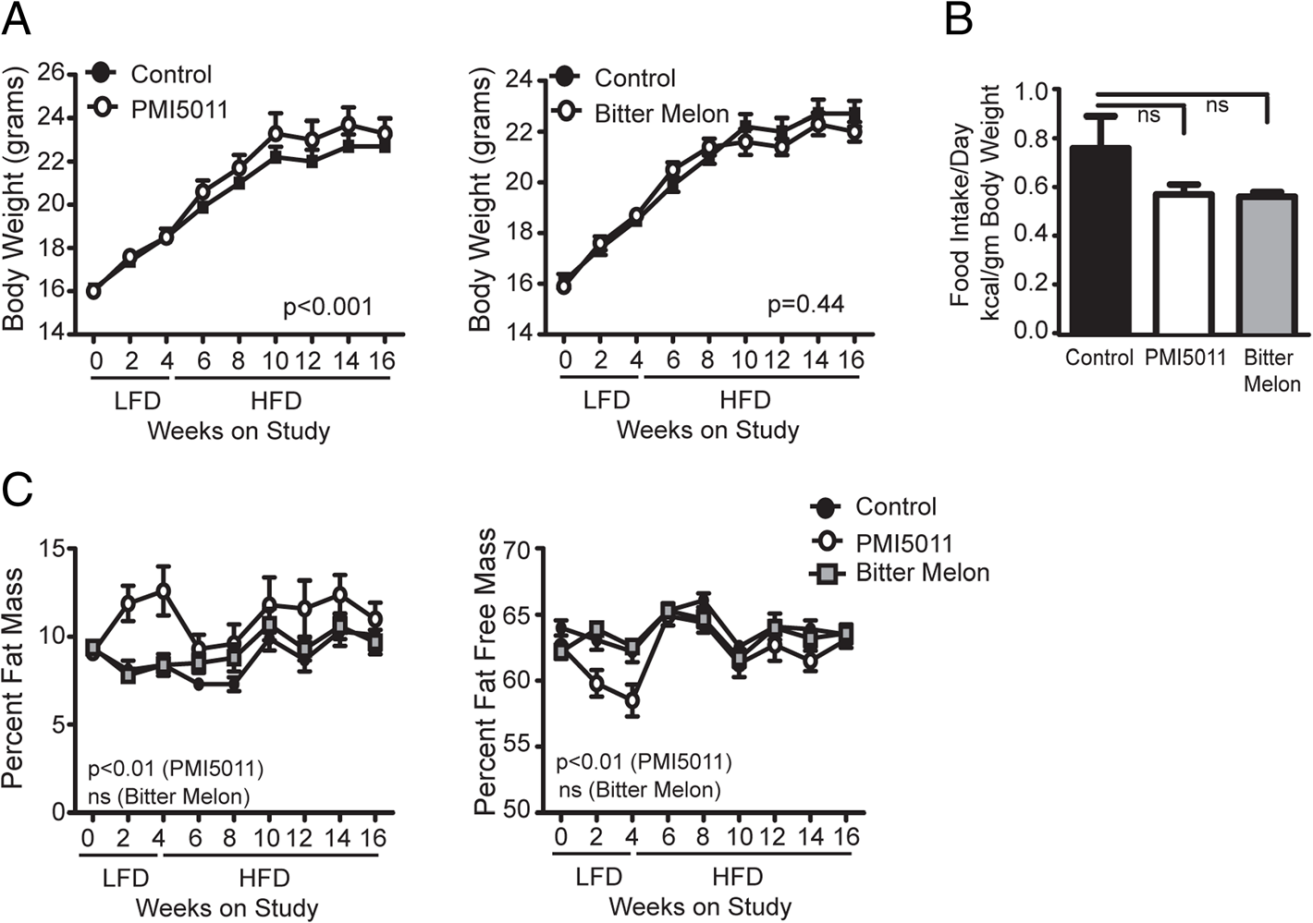
Effects of PMI5011and bitter melon on body composition in female mice: PMI5011 supplementation resulted in a significant increase in body weight due to increased adiposity unrelated to food intake, whereas bitter melon did not affect body weight (**a**). PMI5011 and bitter melon resulted in a non-significant reduction in food intake on a high-fat diet (**b**). An increase in percent fat mass and corresponding decrease in fat-free mass was observed in females supplemented with PMI5011, while bitter melon did not affect fat mass or fat-free mass (**c**). Statistical significance was set at *p* < 0.05, as determined by repeated measures ANOVA. Variability is expressed as mean ± SD. LFD low-fat diet, HFD high-fat diet

A number of previous studies established that the ethanolic extract from *A*. *dracunculus* termed PMI5011 enhances insulin signaling in skeletal muscle and improves insulin sensitivity on a preexisting background of insulin resistance in vitro and in vivo in male mice [22, 24, 25, 48, 49]. To determine if high-fat diet-related insulin resistance can be prevented in female mice by dietary supplementation with either PMI5011 or bitter melon, we carried out a feeding study in female C56BL/6J mice given a LFD alone or supplemented with PMI5011 or bitter melon for 1 month beginning at 4 weeks of age. After 1 month, the diet was switched to a 45% HFD alone or supplemented with PMI5011 or bitter melon and these diets were maintained for 3 months. As shown in Fig. 1a, c, PMI5011 supplementation resulted in a statistically significant increase in body weight due to increased adiposity unrelated to food intake in female mice. Bitter melon supplementation did not affect body weight. Both PMI5011 and bitter melon supplementation resulted in a non-significant reduction in food intake in females on the HFD (Fig. 1b). While PMI5011 supplementation resulted in a significant increase in percent fat mass in females (Fig. 1c), bitter melon supplementation in female mice did not change body weight or body composition (Fig. 1a, c).

### Effect of PMI5011 and bitter melon on energy expenditure and substrate utilization in female mice

**Fig. 2:**
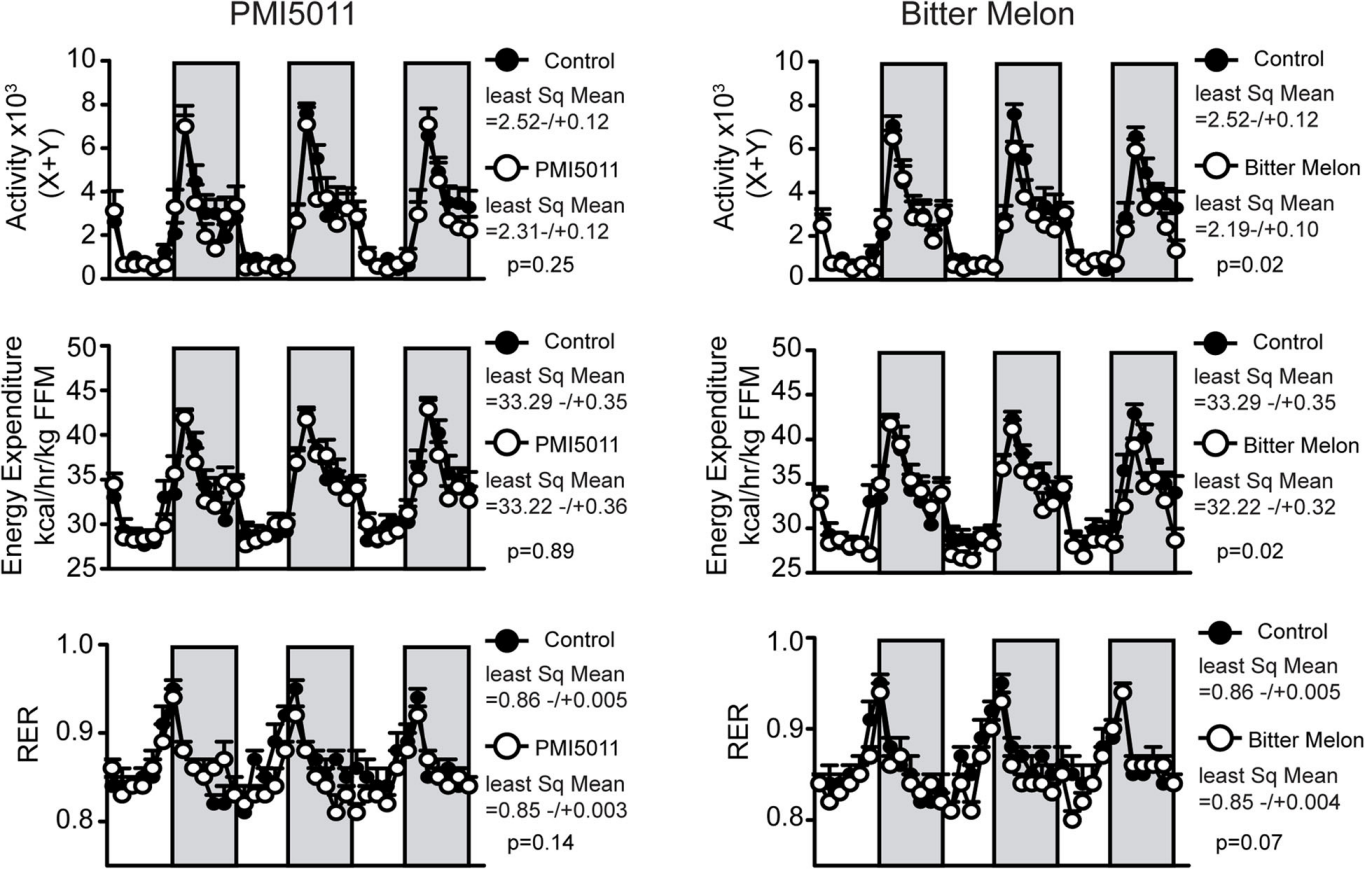
Effects of PMI5011 and bitter melon on energy expenditure and substrate utilization in high-fat-fed female mice: Bitter melon, but not PMI5011, decreased energy expenditure and physical activity. RER was not altered by supplementation with bitter melon or PMI5011. Statistical significance was set at *p* < 0.05, as determined by least squares means analysis. Variability is expressed as mean ± SD

Our data on body weight and adiposity revealed that the female mice did not become obese in response to HFD, a finding that is consistent with other published data [50–52]. We then evaluated activity and energy expenditure to determine which of these factors could account for the resistance to weight gain and adiposity observed in our female mice. PMI5011 did not affect energy expenditure or activity, but we observed decreased activity and energy expenditure in response to bitter melon supplementation (Fig. 2). However, these small decreases in already high levels of activity and energy expenditure were not associated with body weight gain or increased adiposity. Neither botanical supplement significantly altered the respiratory exchange ratio (Fig. 2).

### Carbohydrate and lipid metabolism in response to PMI5011 and bitter melon supplementation

**Fig. 3:**
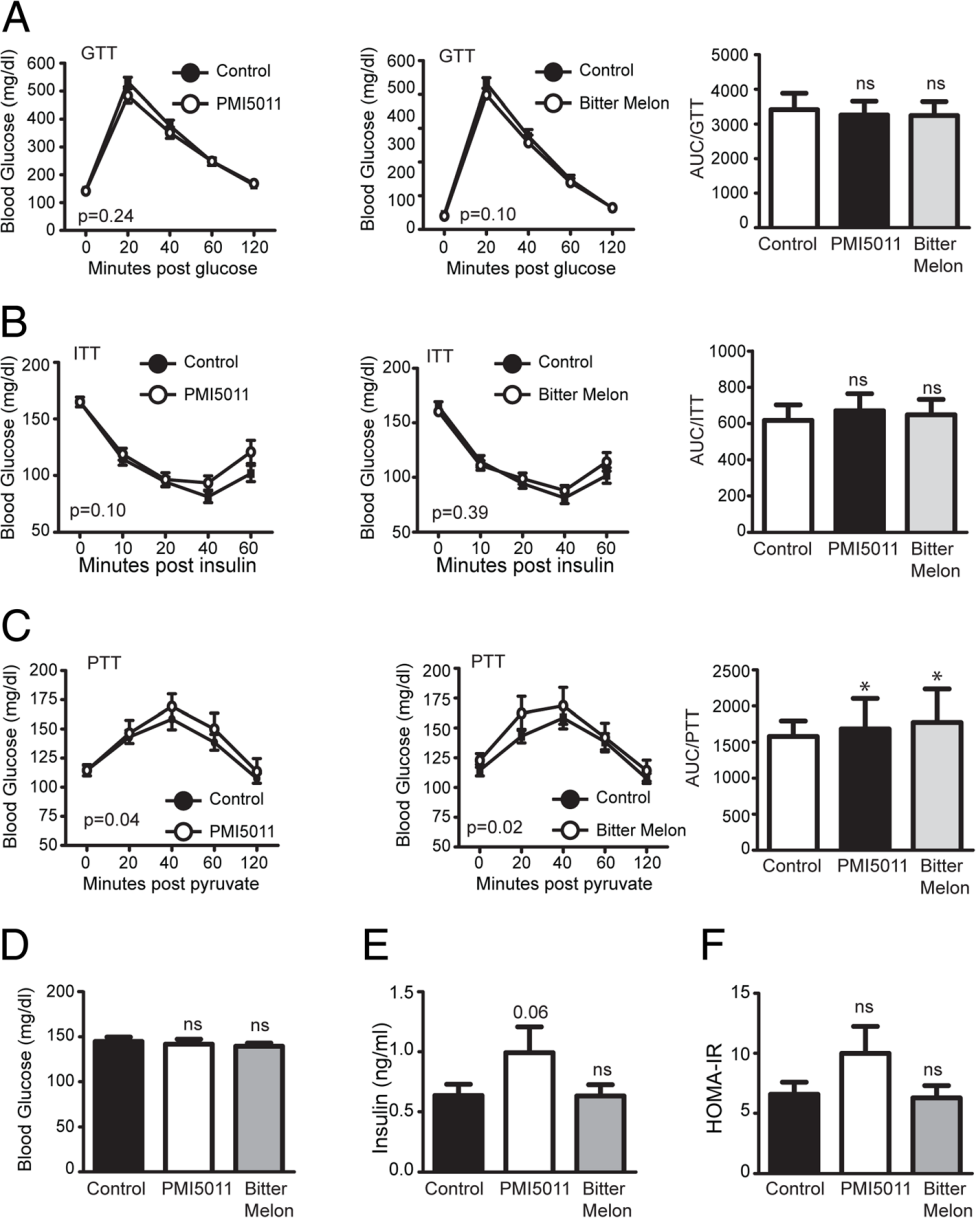
PMI5011 and bitter melon alter carbohydrate and lipid metabolism in high-fat-fed female mice: PMI5011 and bitter melon supplementation do not result in statistically significant alterations in AUC for GTT and ITT (**a**, **b**); however, there was a trend toward higher blood glucose levels at the later time points in the ITT for both PMI5011 and bitter melon (**b**). Pyruvate tolerance tests showed that both bitter melon and PMI5011 increased hepatic glucose production, consistent with increased gluconeogenesis (**c**). Neither PMI5011 nor bitter melon induced statistically significant changes in blood glucose (**d**), although PMI5011 supplementation resulted in a trend (*p* = 0.06) toward elevated fasting insulin (**e**). Statistically significant modulations were not detected in HOMA-IR (**f**), although PMI5011 appears to result in non-significant increase in this parameter. Statistical significance was set at *p* < 0.05, as determined by repeated measures ANOVA or unpaired two-tailed *t* test. Variability is expressed as mean ± SD

We next assayed the effects of PMI5011 and bitter melon on glucose homeostasis and insulin sensitivity in female mice via glucose and insulin tolerance testing along with fasting glucose and insulin levels. Although neither glucose nor insulin tolerance testing shows any statistically significant effect on AUC for glucose for either botanical supplement (Fig. 3a, b), we observed that glucose levels were somewhat higher at the later time points for both PMI5011 and bitter melon in insulin tolerance testing (Fig. 3b). This raised the possibility that the supplements induced alterations in hepatic glucose production, which we assessed by conducting pyruvate tolerance testing. Repeated measures ANOVA indicated that both supplements significantly increased glucose production, consistent with increased gluconeogenesis (Fig. 3c). However, fasting serum glucose and insulin levels indicate that although neither supplement significantly altered blood glucose concentrations, PMI5011 induced an increase in fasting serum insulin (Fig. 3e) that approached statistical significance (*p* = 0.06). Collectively, these data suggest that neither botanical supplement favorably alters glucose homeostasis or insulin sensitivity in female mice challenged with a HFD, with PMI5011 demonstrating a tendency to increase fasting insulin levels despite the lack of a commensurate reduction in fasting glucose. Furthermore, HOMA-IR data suggests that PMI5011 negatively modulates whole-body insulin sensitivity, whereas bitter melon supplementation did not affect HOMA-IR (Fig. 3f).

### Effect of PMI5011 and bitter melon on lipid metabolism in female mice

**Fig. 4:**
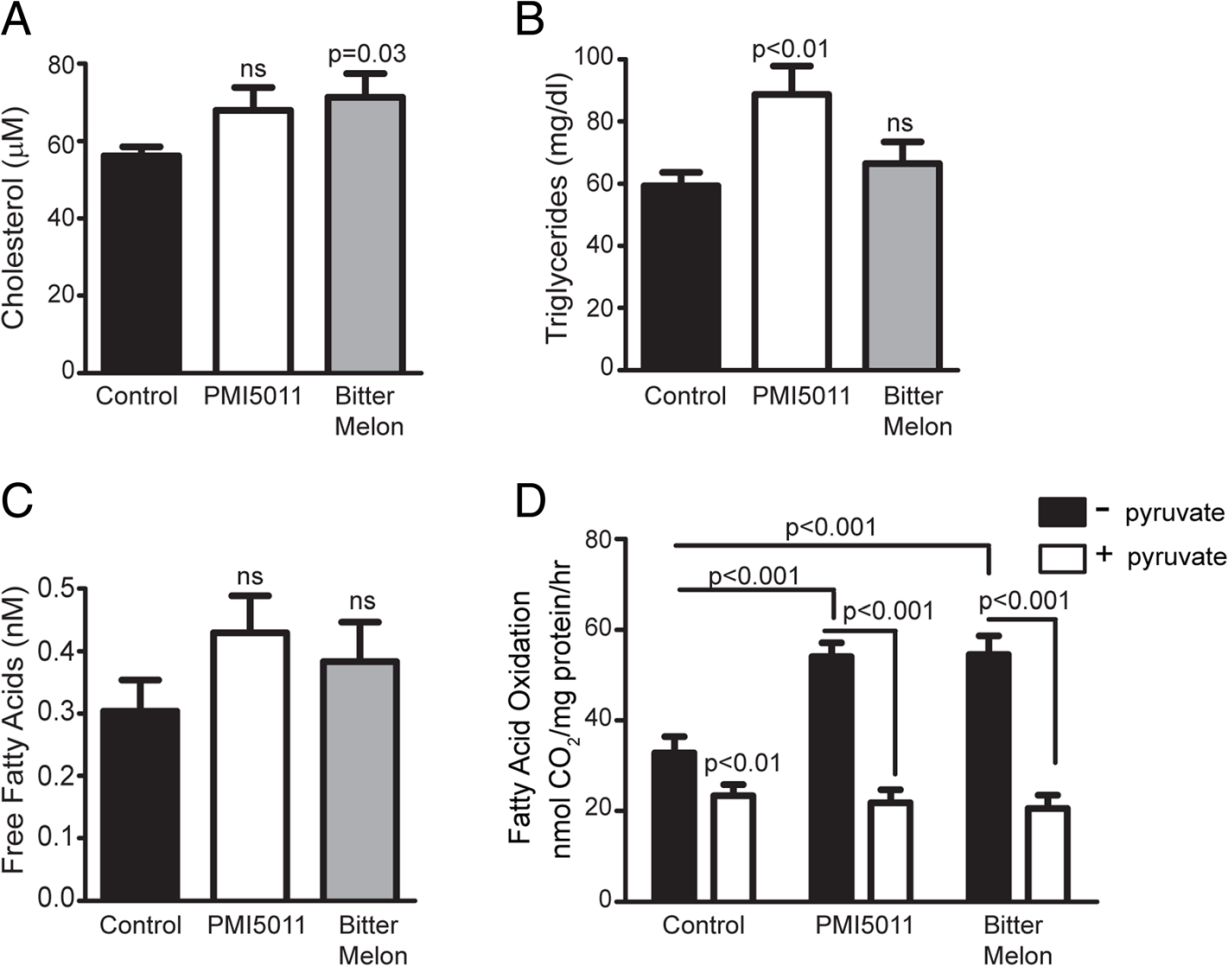
PMI5011 and bitter melon modulate lipid metabolism in high-fat-fed female mice: Bitter melon induced a statistically significant increase in total cholesterol, while PMI5011 modestly increased total cholesterol (**a**). Serum triglycerides were significantly increased by PMI5011, but not by bitter melon (**b**). Both supplements induced non-significant trends toward increased serum free fatty acids (**c**). Mixed gastrocnemius homogenates exhibited an increase in the rate of fatty acid oxidation in response to PMI5011 and bitter melon supplementation (**d**). Fatty acid oxidation rates in mixed gastrocnemius homogenates were suppressed when exposed to pyruvate in the female mice supplemented with PMI5011 or bitter melon, indicating enhanced metabolic flexibility in response to changes in nutrient availability (**d**). Statistical significance was set at *p* < 0.05, as determined by unpaired two-tailed *t* test. Variability is expressed as mean ± SD

To determine the effect of PMI5011 and bitter melon on blood lipids in high-fat-fed female mice, we assayed total cholesterol, serum triglycerides, and free fatty acids. PMI5011 modestly increased total cholesterol while bitter melon induced a statistically significant increase in total cholesterol (Fig. 4a). Serum triglycerides were significantly increased by PMI5011, whereas bitter melon did not appear to have an effect (Fig. 4b). Both botanicals tended to increase serum free fatty acids, although neither effect was statistically significant (Fig. 4c). To further investigate the effects of PMI5011 and bitter melon on lipid metabolism in females, we assessed fatty acid oxidation rates in mixed gastrocnemius homogenates in response to treatment with these botanicals. Results indicate that both supplements increase the capacity of skeletal muscle to utilize lipid as a metabolic fuel source in response to a high-fat diet, as shown by significant increases in fatty acid oxidation rates compared to controls (Fig. 4d). Moreover, both PMI5011 and bitter melon robustly suppress fatty acid oxidation rates in mixed gastrocnemius homogenates exposed to pyruvate ex vivo as a surrogate for glucose oxidation (Fig. 4d). Although carbohydrate metabolism is not assessed by pyruvate-mediated suppression of fatty acid oxidation, the results indicate that both PMI5011 and bitter melon promote metabolic flexibility in the context of high-fat diet consumption.

### Enhanced metabolic flexibility in skeletal muscle in response to PMI5011 and bitter melon supplementation is not mediated by transcriptional regulation

**Fig. 5:**
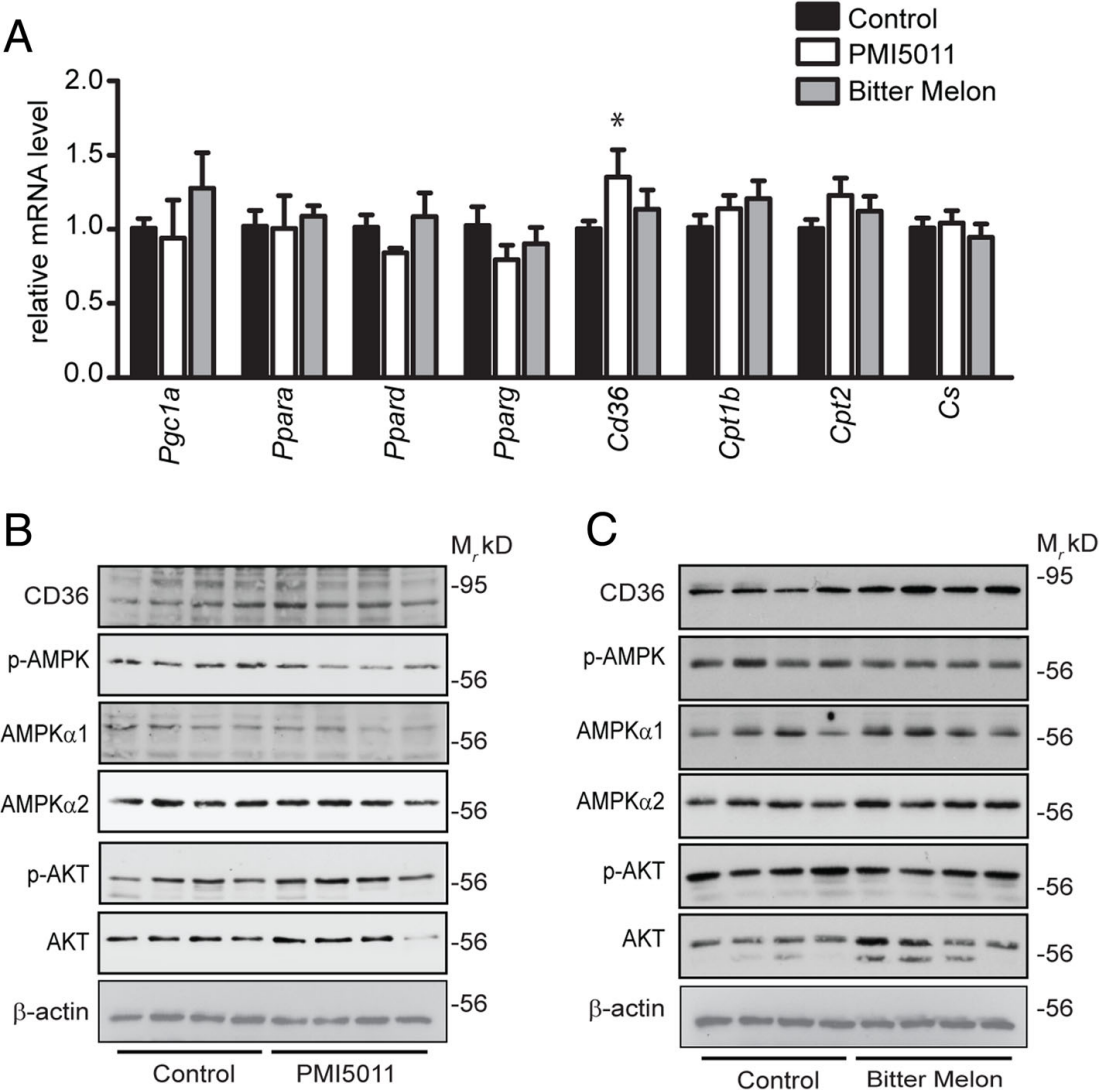
Botanical supplement-induced enhanced metabolic flexibility in skeletal muscle is not mediated by transcriptional regulation: Neither PMI5011 nor bitter melon alters overall patterns of mRNA expression (**a**) or protein abundance (**b**, **c**) in biochemical factors regulating fatty acid metabolism or insulin signaling in high-fat-fed female mice. However, both mRNA and protein abundance of CD36 were increased in the skeletal muscle of mice supplemented with PMI5011 (**a**, **b**). Statistical significance was set at *p* < 0.05, as determined by unpaired two-tailed *t* test. Variability is expressed as mean ± SD

In order to investigate the biochemical mechanisms underlying the improved metabolic flexibility in skeletal muscle observed in female mice, mRNA and protein abundance of a range of factors controlling fatty acid metabolism were assayed by qRT-PCR and immunoblotting, respectively. Overall, neither PMI5011 nor bitter melon induced changes in the gene expression of transcription factors (*Pgc1a*, *Ppara*, *Ppard*, *Pparg*) that regulate fatty acid oxidation or mitochondrial function (*Cpt1b*, *Cpt2*, *Cs*). However, PMI5011 increased mRNA and protein levels of CD36 and bitter melon intake is associated with increased CD36 protein levels, suggesting increased fatty acid uptake in the skeletal muscle of the PMI5011 and bitter melon-supplemented females (Fig. 5a, c). Interestingly, the increased fatty acid oxidation rates with botanical supplementation (Fig. 4d) are not associated with increased activation of AMPK or increased insulin responsiveness as measured by AKT phosphorylation (Fig. 5b, c).

### Effects of PMI5011 and bitter melon on hepatic gene and protein expression

**Fig. 6:**
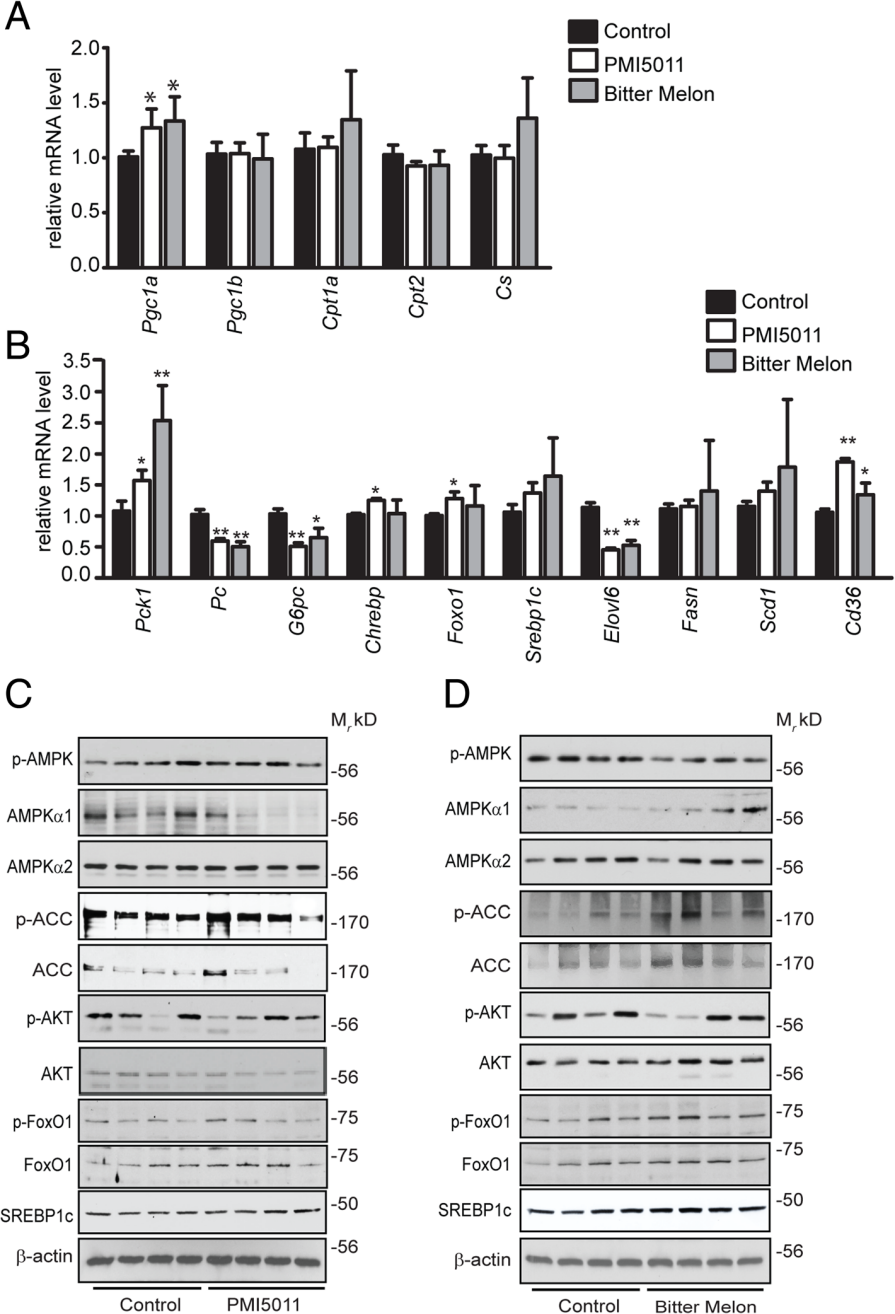
Effects of PMI5011 and bitter melon on hepatic gene and protein expression in high-fat-fed female mice: Both PMI5011 and bitter melon increased mRNA abundance of *Pgc1a*, but not other markers of lipid metabolism (**a**). Markers of de novo lipogenesis are not upregulated while *Cd36* and a marker of gluconeogenesis (*Pck1*) are upregulated (**b**). PMI5011 (**c**) and bitter melon (**d**) do not regulate the levels of proteins involved in lipid metabolism or insulin signaling. Statistical significance was set at *p* < 0.05, as determined by unpaired two-tailed *t* test. Variability is expressed as mean ± SD

The mild elevation in blood glucose in the HFD-fed females (Fig. 3d) and increased blood glucose observed with the pyruvate tolerance test in the botanical-supplemented females (Fig. 3c) prompted us to assay gene and protein expression of factors controlling hepatic glucose and lipid metabolism. Although *Pgc1a* increases, expression of the lipogenic transcriptional regulator *Pgc1b* and markers of mitochondrial function (*Cpt1a*, *Cpt2*, *Cs*) is unchanged (Fig. 6a). De novo lipogenesis (DNL) does not appear to be regulated by either botanical. Although *Chrebp1* and *Foxo1* levels are modestly upregulated by PMI5011, the botanicals do not alter *srebp-1c* expression. Moreover, SREBP-1c target genes that regulate DNL (*Scd1*, *Fasn*) are not increased by the botanicals or, in the case of *Elovl6*, are suppressed (Fig. 6b). In contrast, the gene encoding PEPCK (*Pck1*) is significantly upregulated, consistent with increased gluconeogenesis. However, expression of two other genes regulating gluconeogenesis, *g6pc* and *pc*, is reduced.

To determine if the botanicals altered signaling events controlling hepatic glucose and lipid metabolism, we assayed the phosphorylation status of AMPK, ACC, FoxO1, and AKT as well as the steady state levels of SREBP-1c with PMI5011 (Fig. 6c) or bitter melon (Fig. 6d) supplementation. Hepatic AMPK activity does not increase in response to supplementation with either botanical, as indicated by diminished AMPKα1 protein expression and unaltered ACC phosphorylation. Additionally, acute insulin-dependent AKT phosphorylation is not enhanced by the botanicals in the female mice. Failure of the botanicals to enhance insulin responsiveness is also reflected in the absence of changes in phosphorylation-dependent downregulation of FoxO1. While SREBP-1c is not regulated by PMI5011, the levels trend upward with bitter melon supplementation.

### Effect of PMI5011 and bitter melon on lipid accumulation

**Fig. 7:**
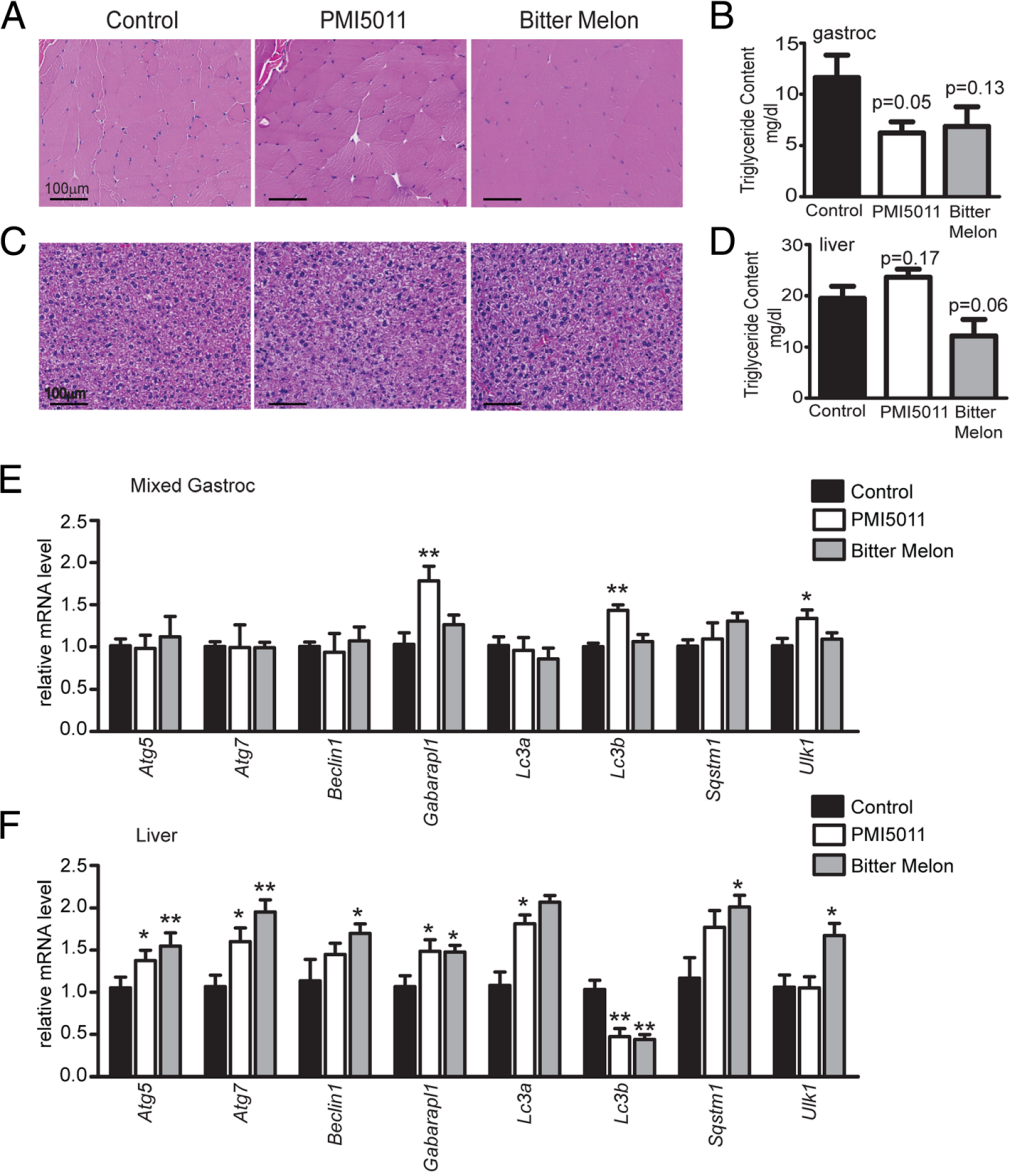
PMI5011 and bitter melon modulate lipid accumulation in high-fat-fed female mice: H&E staining indicates that both PMI5011 and bitter melon induce alterations in lipid accumulation in the mixed gastrocnemius muscle and liver (**a**, **c**). Triglyceride assays showed that PMI5011 induced statistically significant reductions in triglyceride content in mixed gastrocnemius muscle (**b**). Bitter melon resulted in a trend (*p* = 0.06) toward reduction in liver triglyceride accumulation whereas PMI5011 induced a non-significant increase in hepatic triglyceride levels although lipid accumulation was not readily apparent with H&E staining on the females fed a high-fat diet alone (**d**). PMI5011 supplementation induced some transcriptional regulation in a set of autophagic genes in the skeletal muscle and liver, although this effect was not as pronounced as that observed in response to bitter melon supplementation (**e**, **f**). Statistical significance was set at *p* < 0.05, as determined by unpaired two-tailed *t* test. Variability is expressed as mean ± SD

We next assessed lipid content in the skeletal muscle and liver by histological examination. Analysis by hematoxylin and eosin staining indicated that both botanicals induced changes in lipid accumulation in mixed gastrocnemius muscle and liver (Fig. 7a, c). To assess lipid accumulation quantitatively, we performed triglyceride assays on the liver and mixed gastrocnemius samples. Results showed a reduction in lipid content in mixed gastrocnemius that was statistically significant with PMI5011 but not bitter melon (Fig. 7b). Bitter melon supplementation resulted in a non-significant reduction (*p* = 0.06) in triglyceride content in the liver whereas PMI5011 induced a non-significant increase in hepatic triglyceride levels (Fig. 7d) although lipid accumulation was not readily apparent with H&E staining on the females fed a high-fat diet alone (Fig. 7a, c).

Autophagy is an important cellular process that is engaged in response to challenges to energy homeostasis such as fasting and exercise [53]. Moreover, evidence indicates that increased hepatic autophagy is positively associated with an improved serum lipid profile [54] and there is compelling evidence that hepatic lipid storage is regulated by autophagy [55]. Autophagic genes are transcriptionally regulated by nutrient conditions [56]. Thus, we assessed the mRNA levels of a panel of autophagic genes (*atg5*, *atg7*, *beclin1*, *gabarapl1*, *sqstm1*, *ulk1*, *lc3a*, *lc3b*) in the liver and skeletal muscle. We observed a degree of transcriptional regulation in the muscle and liver by PMI5011 (Fig. 7e, f). However, bitter melon induced robust upregulation in autophagic genes in the liver (Fig. 7f). These results are consistent with reduced lipid accumulation in the liver of the bitter melon-fed female mice compared to those supplemented with PMI5011.

## Discussion

Approximately one third of adults in the USA have obesity-related metabolic syndrome, defined by the presence of insulin resistance, dyslipidemia, hypertension, and visceral obesity [57, 58]. Although metabolic syndrome is typically associated with central/visceral obesity in men and post-menopausal women, it is becoming more prevalent in premenopausal women with central obesity associated with increased waist circumference [58]. Thus, it is important to include fertile female rodents in preclinical animal studies designed to test potential therapeutic approaches to combat metabolic syndrome.

In the current study, we sought to determine if dietary supplementation with PMI5011 or bitter melon prior to the onset of high-fat diet-induced obesity prevents development of high-fat diet-related insulin resistance in female C57BL/6 mice. Although the botanicals increase fatty acid oxidation capacity in skeletal muscle, lipid profiles are not improved and early indications of hepatic insulin resistance related to glucose output are observed with botanical supplementation in the female mice. Thus, our results indicate that dietary supplementation with PMI5011 and bitter melon is likely more effective in males than females in preventing high-fat diet-induced insulin resistance. In part, this is due to the female C57BL/6 mouse resistance to high-fat diet-induced obesity or insulin resistance, as found in earlier studies [50–52]. We found that obesity resistance in the female mice is independent of botanical supplementation although dietary intake of PMI5011 increases body weight and percent fat mass in the females compared to the high-fat diet alone. PMI5011-mediated fat accumulation occurs although energy intake and expenditure are comparable to the high-fat diet-fed females. This surprising finding suggests PMI5011 supplementation may increase the efficiency of calorie absorption in the gut, resulting in storage of excess energy in adipose tissue. Interestingly, the rate of body weight gain decreased after 6 weeks on the high-fat diet in all groups and is associated with a striking dissimilarity in energy expenditure due to higher physical activity levels of the female mice compared to previously reported data in male mice [26]. Thus, elevated physical activity may account for resistance to high-fat diet-induced obesity typically observed in the female C57BL/6 mice. It is important to note, however, that in the present study the mice began the high-fat diet at 8 weeks of age. The age of onset of high-fat diet consumption in female C57BL6 mice can be an important factor in determining weight gain [59], and it is possible that the absence of weight gain observed in the females is at least partially accounted for by the age when the high-fat diet was introduced. Nonetheless, sex differences in body weight gain, adiposity, activity, and energy expenditure is a notable finding in the present study and carries implications for future studies aimed at investigating sex as a biological variable in a nutritional context. The data reported herein indicate that sex-related differences in physical activity and energy expenditure can be a determining factor underlying variable responses to dietary intervention and are therefore a major consideration in experimental design and interpretation of results.

Next, we employed several methods to investigate the efficacy of the botanical supplements in modulating insulin sensitivity and glucose homeostasis both systemically and at the individual tissue level. Neither PMI5011 nor bitter melon induced any significant differences in the responses to glucose or insulin tolerance testing, supporting the notion that these botanicals do not enhance glucose disposal in high-fat-fed female mice. This is consistent with the observation that PMI5011 supplementation was associated with a trend toward elevated fasting serum insulin levels despite the absence of any commensurate decrease in fasting glucose. Evaluation of the HOMA-IR, coupled with no increase in skeletal muscle protein kinase B (AKT) activity, further consolidates the interpretation that neither of the botanicals improves whole-body glucose homeostasis in female mice when challenged with a high-fat diet.

We also observed a general pattern of elevated plasma lipid levels in the female mice associated with both PMI5011 and bitter melon supplementation that was absent in previous studies of high-fat-fed male mice [26]. However, we note an absence of any correlation between the botanical-mediated increase in plasma fatty acid levels and adiposity or insulin sensitivity in females, consistent with higher fatty acid levels in women independent of fat mass when compared to men [60]. Importantly, skeletal muscle contributes to whole-body glucose disposal to a greater degree than any other tissue type and insulin resistance related to dysregulation of lipid metabolism in skeletal muscle is a defining characteristic of metabolic syndrome and type 2 diabetes [61]. The upward trend in lipid levels in response to the botanicals is met by an increased capacity in the females to use fatty acids as a fuel source in skeletal muscle. The botanicals promoted a “metabolically flexible” phenotype in skeletal muscle that was reflected in the trend toward a lower respiratory exchange ratio consistent with increased ability to use fatty acids as a fuel source while consuming a high-fat diet. However, analysis of genes and proteins involved in the regulation of lipid metabolism indicates enhanced metabolic flexibility is largely unrelated to transcriptional regulation in skeletal muscle, although PMI5011 induced a significant increase in *cd36* mRNA expression. This finding raises the possibility that CD36-mediated fatty acid uptake in skeletal muscle in the females contributes to enhanced fatty acid oxidation with dietary intake of PMI5011. This mechanism may also influence fatty acid metabolism in skeletal muscle of the bitter melon-supplemented females as CD36 protein levels are increased with either botanical.

However, botanical supplementation in the females may be associated with increased hepatic glucose output as indicated by higher glucose levels with botanical supplementation at the late time points on the insulin tolerance test, elevated glucose with pyruvate tolerance testing and increased mRNA levels of *pck1* in the liver with botanical supplementation. It is possible that the increased glucose levels at the later time points with botanical supplementation in the insulin tolerance test is due to counterregulatory mechanisms against hypoglycemia since these mechanisms occur in male mice at 80 mg/dl glucose [62], a level observed in the female mice. Even so, physiological adaptation to defend glucose levels seems an unlikely explanation for the increased glucose output upon pyruvate tolerance testing or the elevated hepatic *pck-1* mRNA levels at the end of study.

Insulin mediates hepatic glucose output by regulating transcription of the rate-determining enzymes in gluconeogenesis: cytosolic phosphenolpyruvate carboxykinase, encoded by *Pck1* and the catalytic subunit of glucose-6-phosphatase encoded by *G6pc*, which is also rate-limiting in glycogenolysis (reviewed in [63]). Insulin-dependent phosphorylation of AKT suppresses *Pck1* and *G6pc* via regulation of FoxO1 and PGC-1α activity. At the same time, insulin signaling is associated with increased hepatic de novo lipogenesis (DNL) via ChREBP and SREBP-1c transcriptional activity [64]. Botanical supplementation does not significantly affect expression of key transcriptional regulators of DNL at the gene or protein level, and expression of the fatty acid elongase gene *elovl6*, a target of SREBP-1 that catalyzes long chain fatty acid formation, is suppressed, supporting decreased DNL. However, the effect of PMI5011 and bitter melon on the balance between glucose output and lipid synthesis in the liver appears to be complex as triglyceride levels trend upward with PMI5011 and are unchanged with bitter melon.

Pathway-selective hepatic insulin resistance is proposed to explain the failure of insulin to suppress glucose production but support lipogenesis and liver fat accumulation [65]. However, hepatic triglycerides are primarily derived from nonesterified fatty acids (NEFAs) rather than DNL [66] and re-esterification of NEFAs is driven by substrate availability independent of insulin signaling or transcriptional changes related to lipogenesis [67]. Our data is consistent with a model of botanical-mediated early pathway-selective insulin resistance in the liver characterized by a failure of insulin signaling to suppress glucose output. Unlike previous studies in male mice, the botanicals do not enhance insulin signaling in the females. Thus, DNL is not increased with dietary botanical supplementation when compared to the HFD alone. Increased uptake of NEFAs with the high-fat diet is supported by the increased *Cd36* expression and the modest upregulation of triglycerides, primarily for PMI5011 supplementation. Thus, increased hepatic glucose output in response to the botanicals raises concerns about possible early adverse effects of the botanicals on glucose metabolism in the high-fat-fed females.

Despite evidence suggesting that supplementation with PMI5011 and bitter melon may adversely affect hepatic function, we found that there were some advantageous effects of the supplements on ectopic lipid accumulation in the skeletal muscle and liver. In the skeletal muscle, PMI5011 significantly reduced triglyceride accumulation while bitter melon induced a downward trend, consistent with enhanced fatty acid oxidation in the skeletal muscle. In the liver, dietary intake of bitter melon decreased triglyceride content to a level that approached statistical significance (*p* = 0.06) while PMI5011 was associated with increased hepatic triglycerides. Remarkably, modulation of lipid accumulation in these tissues was closely associated with transcriptional regulation of autophagic genes with the most robust response elicited by bitter melon in the liver. Transcriptional regulation of autophagy is regulated by more than 20 transcription factors [56]. Our findings are the first to identify bitter melon-mediated transcriptional regulation of hepatic autophagy. Interestingly, upregulation of gamma-aminobutyric acid receptor-associated protein-1 (*Gabarapl1*), which encodes a ubiquitin-like protein associated with autophagic vesicles, was first identified as an estrogen-regulated gene [68]. *Gabarapl1* is most robustly upregulated in skeletal muscle in the PMI5011-supplemented females where estrogen plays an important role in skeletal muscle quality in males [69] as well as females.

## Conclusion

The more pronounced effect of PMI5011 supplementation on improving insulin sensitivity and reducing ectopic lipid accumulation in the skeletal muscle and liver previously reported in males on a high-fat diet compared to females indicates there are sex-related differences in the potential benefits of dietary intake of PMI5011 in preventing risk factors for metabolic syndrome. Alternatively, the absence of any evidence of high-fat diet-induced obesity or insulin resistance in the females raises the possibly that PMI5011 and bitter melon are effective only in the presence of obesity-related insulin resistance as observed in our previous animal studies in male mice [25, 26] as well as in vitro models of insulin resistance in skeletal muscle [22, 24, 48]. In that case, PMI5011 and bitter melon may be effective in females that develop insulin resistance with obesity. Although our current study does not differentiate between these two possibilities, our data supporting early selective hepatic insulin resistance related to glucose production with PMI5011 or bitter melon dietary supplementation underscores the potential for untoward effects of botanical supplementation in females and the importance of considering sex-related variations in metabolism and metabolic responses to dietary supplementation.

## Additional files

Additional file 1: Antibody Information. (PDF 13 kb)

Additional file 2: Supporting Information Related to Gene Expression Analysis. (PDF 81 kb)

## Abbreviations

ACC: Acetyl-CoA carboxylase
AKT: Protein kinase B
AMPK: AMP-activated protein kinase
CPT-1: Carnitine palmitoyltransferase I
DNL: De novo lipogenesis
HFD: High-fat diet
HOMA-IR: Homeostatic model assessment for insulin resistance
IL-6: Interleukin-6
IRS-1: Insulin receptor substrate-1
JNK: c-Jun N-terminal kinase
LFD: Low-fat diet
MetS: Metabolic syndrome
PEPCK: Phosphoenolpyruvate carboxykinase
SOCS-3: Suppressor of cytokine signaling-3
TNF-α: Tumor necrosis factor-alpha
