# Supplementary material for: Potential adverse effects of botanical supplementation in high-fat-fed female mice
Source: Biol Sex Differ. 2018 Sep 12;9:41. doi: 10.1186/s13293-018-0199-1 (PMC6134698; doi:10.1186/s13293-018-0199-1)
Supplement: Supplementary file 1 — Antibody Information. (PDF 13 kb) [file 13293_2018_199_MOESM1_ESM.pdf]

Fuller *et al*
Additional File 1_Antibody Information

Primary Antibody	Type	Application	Supplier	Catalog Number
AMPKa1	Rabbit polyclonal	Western blot	Cell signaling	2795
AMPKa2	Rabbit polyclonal	Western blot	Millipore	07-363
AMPK-p Thr132	Rabbit polyclonal	Western blot	Cell signaling	2535
PGC1a	Rabbit polyclonal	Western blot	Abcam	ab54481
FoxO-1	Rabbit polyclonal	Western blot	Abcam	ab39670
p-FoxO1	Rabbit polyclonal	Western blot	Abcam	ab131339
SREBP-1c	Mouse monoclonal	Western blot	Santa Cruz	sc-13551
CD36	Rabbit monoclonal	Western blot	Cayman Chemical	100011
AKT	Mouse monoclonal	Western blot	Cell signaling	2920
AKT-p Ser473	Mouse monoclonal	Western blot	Cell signaling	4051
ACC	Rabbit polyclonal	Western blot	Cell signaling	3662
ACC-p Ser79	Rabbit polyclonal	Western blot	Millipore	07-303
beta-actin	Rabbit polyclonal	Western blot	Bethyl Laboratories	A300-491A
