# Supplementary material for: Potential adverse effects of botanical supplementation in high-fat-fed female mice
Source: Biol Sex Differ. 2018 Sep 12;9:41. doi: 10.1186/s13293-018-0199-1 (PMC6134698; doi:10.1186/s13293-018-0199-1)
Supplement: Supplementary file 2 — Supporting Information Related to Gene Expression Analysis. (PDF 81 kb) [file 13293_2018_199_MOESM2_ESM.pdf]

Fuller *et al*
Additional File 2_Supporting Information Related to Gene Expression Analysis (All the primers were from IDT)

Gene ID	Gene Name	Accession #	Sequence -Forward	Sequence -Reverse
PGC1 alpha	peroxisome proliferative activated receptor, gamma, coactivator 1 alpha	NM_008904	AGCCTCTTTGCCCAGATCTTC	CCATCTGTCAGTGCATCAAATGA
PGC1 beta	PPARGC1B (PGC1b) PPARG coactivator 1 beta	NM_133249.2	TCCAGAAGTCAGCGGCCT	CTGAGCCCGCAGTGTGG
PPAR alpha	Peroxisome proliferator activated receptor alpha	NM_011144	CCTCAGGGTACCACTACGGAGTT	TCGCCGAAAGAAGCCCTTA
PPAR gamma	Peroxisome proliferator activated receptor gamma	NM_001127330	CACAATGCCATCAGGTTTGG	GCTGGTCGATATCACTGGAGATC
PPAR delta	Peroxisome proliferator activator receptor delta	NM_011145	CAAGTTCGAGTTTGCTGTCAAG	GTGTCTGGAGTGTTGTGAGTAG
Chrebp1/Mlxipl	Carbohydrate response element binding protein	NM_021455.5	GAAACCTGAGGCTGTCATCCT	CGTGGTATTCGCGCATCA
AMPK alpha 1	Protein kinase, AMP-activated, alpha 1 catalytic subunit (Prkaa1)	NM_001013367	TGATGCCGAAGCTCAAGGA	GGGAGGTGACAGATGAGGTAAGG
AMPK alpha 2	Protein kinase, AMP-activated, alpha 2 catalytic subunit (Prkaa2)	NM_178143	TCGCAGACAGCCCCAAAG	TTGGGCTTCGTTGTGTTGAG
G6Pase/g6pc	Glucose-6 Phosphatase catalytic subunit	NM_008061.4	CGAGGAAAGAAAAAGCCAAC	CAAGGTAGATCCGGGACAGA
Gck	Glucokinase	NM_010292	GCT GGT ACG ACT TGT GCT G	TGG ACA CGC TTT CAC AGG
Elovl6	Elongation of Long Chain Fatty Acids, Family Member 6	NM_130450.2	CCCGAACTAGGTGACACGAT	TACTCAGCCTTCGTGGCTTT
Srebp-1/Srebf1	Sterol response element binding transcription factor-1c, SREBP-1, ADD-1	NM_011480	ATC GCA AAC AAG CTG ACC TG	AGA TCC AGG TTT GAG GTG GG
CPT1b	Carnitine palmitoyltransferase 1b	NM_009948	CCCGAGCAGTGCCGGGAAGC	GAAATGAGCCAGCTGTAGGG
CPT2	Carnitine palmitoyltransferase 2	NM_009949	TGCCCAGGCTGCCTATCCCTAAACT	GCTCCTTCCCAATGCCGTTCTCAAAAT
Scd-1	Stearoyl-Coenzyme A Desaturase 1	NM_009127.4	GCTGGGCAGGAACTAGTGAG	GGTAGGGAGGATCTGGAAGC
Cs	Citrate synthase	NM_026444	CGGGAGGGCAGCAGTATCGG	ACCACCCTCATGGTCACTATGGATG
pck1	Phosphoenolpyruvate carboxykinase 1	NM_011044.3	GACAGTCATCATCACCCAAGAG	GGTTAGTTATGCCCAGGATCAG
pc	Pyruvate Carboxylase	NM_012744	CCTGCTCGTCAAAGTCATTGC	ACACCTCGGACACGGAACTC
cd36	Cluster of differentiation 36	NM_001159555	GAAGCATTAAAGAATCTGAAGAGACC	GGATTTGCAAGCACAATATGAAATC
FOXO1	Forkhead box protein O1	NM_019739	CCCGTCCTAGGCACGAACT	ACGCGCCCAGAACTTAACTTC
FASN	Fatty acid synthase	NM_007988.3	GTGTGGAAAGCTGAAGGATCTC	TCTCGGGATCTCTGCTAAGG
SQSTM1	Sequestosome 1	NM_011018	GTGGTGGGAACTCGCTATAAG	ATCTGGGAGAGGGACTCAAT
LC3A	Microtubule-associated protein 1 light chain 3 alpha	NM_025735	TGTCCTGGATAAGACCAAGTTTC	GGTGTCACATCTCTGCCTAATC
LC3B	Microtubule-associated protein 1 light chain 3 beta	NM_026160	CCACCAAGATCCCAGTGATTA	CTACAACACCAGACCTGCTTAG
Beclin 1	Beclin 1, autophagy related	NM_019584	CAGGAACTCACAGCTCCATTAC	CTGCTCACTGTCATCCTCATTC
ULK1	Unc-51 like kinase 1	NM_009469	GTCTACCTGGTCATGGAGTATTG	CATCGTAGTGCTGGGACATAA
Gabarapl1	Gamma-aminobutyric acid (GABA) A receptor-associated protein-like 1	NM_020590	TTCTTTACCTGGTGGTCCTTTAC	GGTCCGGGTATTTCTTCCTAATC
ATG3	Autophagy related 3	NM_026402	CCGGTCCTCAAGGAATCAAA	GACTCTATAGCCTCTCCACTACA
ATG5	Autophagy related 5	NM_001314013	TCAACCGGAAACTCATGGAATA	GGAGGACACACTCTTTCAATCT
ATG7	Autophagy related 7	NM_028835	GCCCTGCCCTACTTCTTATTC	GCCTCCTTTCTGGTTCTTCTC
ATG14	Autophagy related 14	NM_172599	GAGCTCACCTCCATCATATTCC	ACTCCAGGGTCCACAAATTC
